# Magnetite Nanoparticles as Solar Photo-Fenton Catalysts for the Degradation of the 5-Fluorouracil Cytostatic Drug

**DOI:** 10.3390/nano12244438

**Published:** 2022-12-13

**Authors:** Lorena T. Pérez-Poyatos, Sergio Morales-Torres, Francisco J. Maldonado-Hódar, Luisa M. Pastrana-Martínez

**Affiliations:** NanoTech—Nanomaterials and Sustainable Chemical Technologies, Department of Inorganic Chemistry, Faculty of Sciences, University of Granada, ES18071 Granada, Spain

**Keywords:** iron catalyst, cytostatic drug, Fenton reaction, photo-Fenton process, solar radiation

## Abstract

Heterogeneous catalysts based on magnetite nanoparticles, Fe_3_O_4_, were prepared by the chemical coprecipitation method using iron (III) chloride as a salt precursor. The physicochemical properties of the nanoparticles were determined by different techniques and the efficiency was evaluated for the degradation of the cytostatic drug, 5-fluorouracil (5-FU), in aqueous solution by photo-Fenton process under simulated solar radiation. The most influential parameters, namely pH of the solution, catalyst load, H_2_O_2_ dosage, and use of radiation, were studied and optimized in the degradation process. The optimal conditions to achieve a 100% degradation of 5-FU (10 mg L^−1^) and a high mineralization degree (76%) were established at the acidic pH of 3.0, 100 mg L^−1^ of catalyst loading, and 58 mM of H_2_O_2_ under simulated solar radiation. The contribution of iron leaching to the catalyst deactivation, the role of the dissolved iron ions on homogenous reactions, and the stability of the catalyst were assessed during consecutive reaction cycles.

## 1. Introduction

Currently, the access to drinking water could be threatened by the uncontrolled dumping of harmful substances created by modern society, including the growing industrial and agricultural discharges. These spillages are capable of causing harm not only to humans but also to the environment in general [1]. In addition, new contaminants of emerging concern (CECs) have been found in urban wastewaters and even in drinking water [2,3] at low concentrations (μg L^−1^), which is an indicator of their great persistence. Their occurrence in the environment has been more recently investigated due to the development of more sensitive analytical methods.

The progressive ageing of the population and the frequent exposure to toxic agents have significantly increased the number of cancer diagnoses, leading to an increase in the consumption of antitumor, cytostatic, or antineoplastic drugs, whose occurrence has been detected in urban wastewater treatment plants (UWWTPs) [4]. Currently, it is estimated that there are more than 100 antineoplastic drugs on the market and this number is expected to grow due to the high demand for these compounds [5]. 5-fluorouracil (5-FU) is a cytostatic antitumor compound that is widely used in Europe [6] for the treatment of breast, colorectal, nasopharyngeal, laryngeal, and oral cavity cancers, and is administered by intravenous route [7]. Its mechanism of action is based on the inhibition of DNA synthesis and its replication by inhibiting the thymidylate synthase enzyme [8]. In recent studies, it has been confirmed that around 15–30% of this compound is excreted without being metabolized [9,10]. Nevertheless, in general, UWWTPs do not have a real capacity to degrade these CECs [11,12].

Among the different advanced oxidation processes (AOPs), the Fenton reaction constitutes a promising technology for the treatment of water and wastewater containing nonbiodegradable compounds [13]. The advantages of the Fenton process are based on: (i) low cost; (ii) low toxicity for the environment; (iii) the use of reagents that are easy to manage and store; (iv) highly efficient degradation; and (v) mild reaction conditions at atmospheric pressure and room temperature [6,14,15], which make it an ideal method for the degradation of organic contaminants. Furthermore, it can also be performed either homogeneously or heterogeneously [15,16] by means of the main reactions shown in Equations (1)–(3):

H_2_O_2_ + Fe^2+^ → HO^•^ + HO^−^ + Fe^3+^ (1)

H_2_O_2_ + Fe^3+ ^ → Fe^2+^ + H^+^ + HO_2_^•^ (2)

HO^•^ + organic compounds → intermediates → CO_2_ + H_2_O (3)

However, the homogeneous Fenton process presents some drawbacks, such as the presence of metals in the solution which entail a harmful effect and should be carefully precipitated after the Fenton process, before discharging the treated solutions. Moreover, the formation of iron sludge should be avoided during the homogenous Fenton treatment in such a way that the reaction presents a strong dependence on the pH of the solution. Maintaining a pH of 3 is required to avoid the precipitation of Fe(OH)_3_ and the dissociation of hydrogen peroxide into molecular oxygen and water, which take place at higher pH values [17].

To overcome these problems, the Fenton process is progressively heterogenized and the pH range could be broadened to near-neutral pH values [6,18,19]. One of the most-used catalysts in the heterogeneous process is magnetite, Fe_3_O_4_, wherein both oxidation states of iron are present in the solid, along with another phases, such as FeTiO_3_ and MnFe_2_O_4_, or even binary materials, such as Fe_3_O_4_/MoS_2_ or Fe_3_O_4_/TiO_2_ [20,21]. This latter catalyst allows the Fenton process to efficiently combine with photocatalysis, which considerably enhances the contaminant degradation [20]. Thus, the Fenton process can be performed in darkness or in the presence of radiation, known as photo-Fenton, which benefits the radical formation and, therefore, the degradation as a whole. On the other hand, it also promotes the reduction of Fe^3+^ ions to Fe^2+^ ions, speeding up the reaction two and the process in general [9,22]. The challenges in the remediation of anticancer drugs were recently reviewed [23], with some studies being focused in the oxidation of 5-FU by using the homogeneous photo-Fenton (UV) process and analyzing the operational parameters for achieving good conversion values [9]. In this work, the catalytic performance of Fe_3_O_4_ nanoparticles as heterogeneous Fenton and photo-Fenton catalysts using solar radiation is presented. The influence of different operational parameters, namely pH of the solution, catalyst loading, H_2_O_2_ dosage, and use of radiation, were deeply studied and optimized to achieve the maximum 5-FU removal at low iron-leaching levels.

## 2. Materials and Methods

### 2.1. Synthesis of Fe_3_O_4_ Nanoparticles

Iron (II, III) oxide (Fe_3_O_4_) magnetic nanoparticles were synthesized following a coprecipitation method in alkaline medium, adapting methodologies described in the literature [24,25]. Both chemicals and synthesis details are summarized in the Supplementary Information. The sample was labelled as Fe_3_O_4_.

### 2.2. Characterization Techniques

The prepared catalyst was characterized by N_2_ adsorption–desorption at −196 °C, X-ray diffraction (XRD), point of zero charge pH (pH_PZC_), scanning electron microscopy (SEM), transmission electron microscopy (HRTEM), and optical properties according to the procedures detailed in the Supplementary Materials Section.

### 2.3. Degradation Study by Solar Photo-Fenton Process

The catalytic activity of the samples was evaluated for the degradation of a 10 mg L^−1^ aqueous solution of 5-FU under simulated solar radiation. The photocatalytic experiments were performed in a Cofomegra SolarBox system (Milano, Italy) equipped with a Xenon arc lamp (1500 W) with outdoor UV glass filters, cutting the transmission of wavelengths below 280 nm. The amount of irradiance entering the photoreactor was equal to 40 W m^−2^. The experiments were performed at varying pHs (from 3.0 to 11.0, by adding H_2_SO_4_ or KOH solutions, respectively), catalyst loads (i.e., 50, 100, and 150 mg L^−1^), and H_2_O_2_ dosages (15, 30, 58, and 82 mM). Additional details are summarized in the Supplementary Material Section.

## 3. Results and Discussion

### 3.1. Materials Characterization

**Figure 1:**
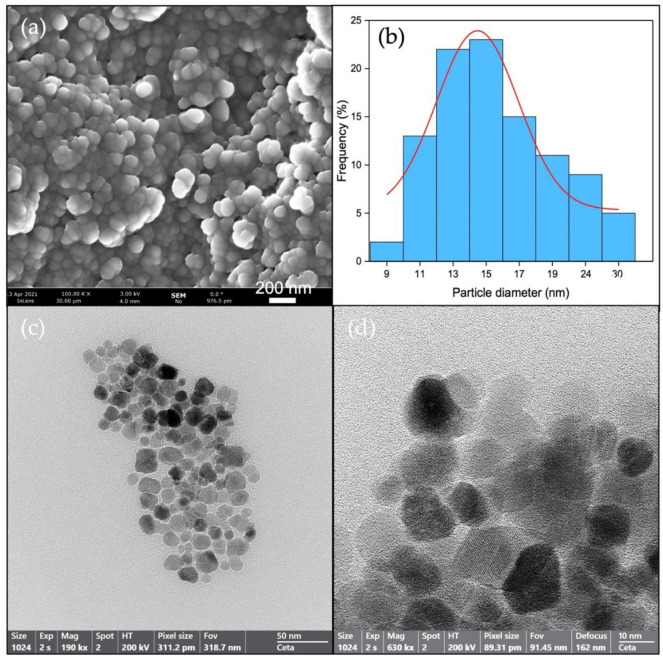
Morphology of Fe_3_O_4_ nanoparticles: (**a**) SEM and (**c**,**d**) TEM micrographs; (**b**) curve distribution of particle size by TEM.

The morphology of the materials studied by SEM and TEM (Figure 1) seemed to consist of clusters of Fe_3_O_4_ nanoparticles with sphere-like morphology. The particle size of these nanoparticles was estimated by analyzing several TEM images using the software ImageJ. The obtained curve distribution is shown in Figure 1b. Particle size followed a Gaussian distribution, with a medium-size particle being around 14.7 nm, although smaller and larger particles were also formed through the coprecipitation method.

**Figure 2:**
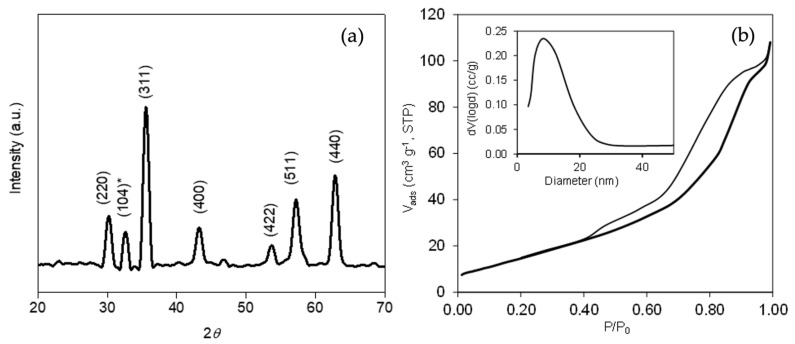
(**a**) XRD pattern of catalyst, assigned to the Fe_3_O_4_ iron phase (JCPDS 85-1436) and (**b**) N_2_-adsorption isotherm and (inset) pore size distribution (PSD).

The crystallographic characteristics of the sample were analyzed by XRD (Figure 2a). The main peaks observed at 30.2°, 35.58°, 43.22°, 53.66°, 57.24°, and 62.8° corresponded, respectively, to the (220), (311), (400), (422), (511), and (540) diffractions of magnetite (Fe_3_O_4_) (JCPDS 85-1436) [26,27]. However, the peak shown at 32.62° was assigned to the (104) plane of hematite (α-Fe_2_O_3_), which means that the synthesized catalyst could contain a certain percentage of this structure [28,29]. The crystallite size estimated from the Scherrer equation was around 13.7 nm, which is in agreement with the size distribution obtained from the TEM images.

Textural characteristics were obtained by N_2_ physisorption. The corresponding adsorption isotherms and pore size distributions (PSDs) are shown in Figure 2b. The amount of N_2_ adsorbed at low relative pressure was very low, denoting the absence of microporosity in the metallic oxide. Nevertheless, the N_2_ adsorption increased with increasing relative pressure (P/P_0_), forming a marked hysteresis loop during desorption, which denoted the formation of mesopores as interstitial voids between the Fe_3_O_4_ nanoparticles. The PSD obtained by the BJH method provided a monomodal mesopore distribution, with a medium pore size of 8.3 nm (Figure 2b, inset), while the BET surface area (S_BET_) and total pore volume (V_T_) were 44 m^2^ g^−1^ and 0.144 cm^3^ g^−1^, respectively.

Regarding the chemical properties, the Fe_3_O_4_ catalyst presented a pH_PZC_ of 3.8, showing an acidic nature similar to that reported in other works [30,31]. This fact indicates that, at operational conditions, pH = 3.0 (i.e., pH < pH_PZC_) and, consequently, that the nanoparticles should present a moderate positive charge associated with the protonation of the –FeOH surface groups to FeOH_2_^+^ [30].

**Figure 3:**
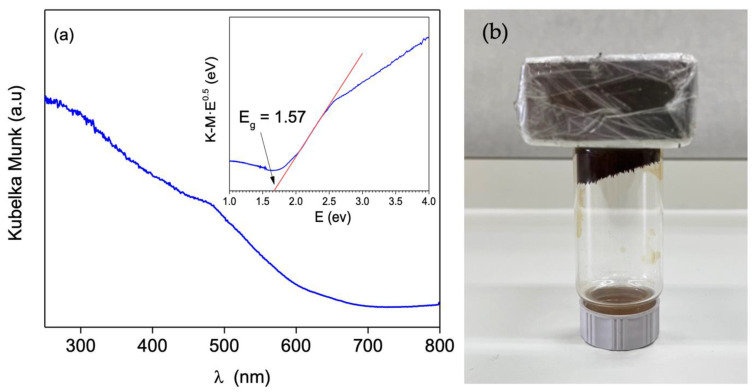
(**a**) UV-Vis spectra and (inset) Tauc’s plots vs. energy (eV) of Fe3O4 sample and (**b**) picture showing magnetic properties.

The optical properties of Fe_3_O_4_ were determined using the diffuse reflectance (DR) UV-Vis spectra, expressed in terms of Kulbelka–Munk equivalent absorption units, as shown in Figure 3a. The observed absorption spectrum revealed a strong absorption in the UV-Vis range, with a major absorption peak at around 295 nm. Tauc’s plots were used to calculate a band-gap (Eg) of 1.57 eV for Fe_3_O_4_ nanoparticles (Figure 3a inset), which varied in value according to the particle size obtained. In our case, the obtained band-gap value was in agreement with the results obtained in the literature, considering a particle size of around 14 nm [32,33].

Furthermore, the magnetic properties are evidenced by the fact that the Fe_3_O_4_ sample was strongly attracted by a magnet (Figure 3b). This aspect is remarkably interesting because the material might be easily recovered by magnetic separation.

### 3.2. Degradation of 5-FU by Solar Photo-Fenton Process

**Figure 4:**
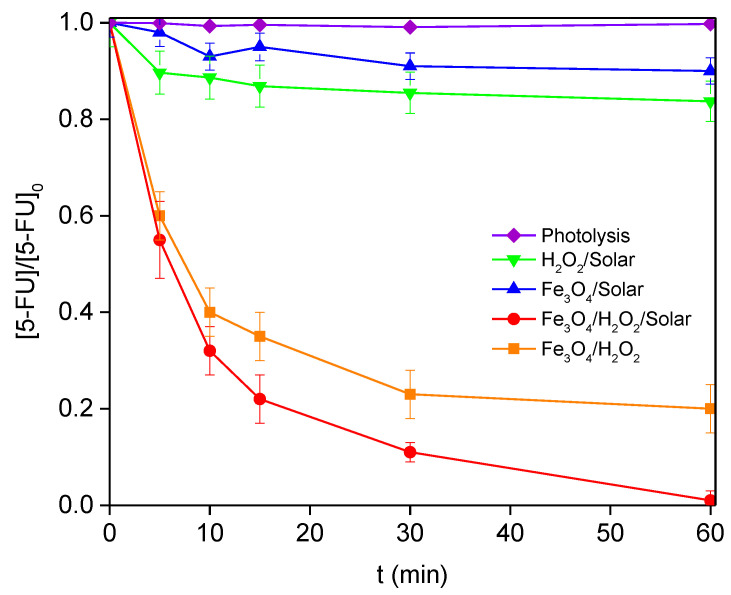
Degradation of 5-FU in the presence/absence of: solar radiation, H_2_O_2_, and heterogeneous catalyst (pH = 3, 58 mM of H_2_O_2_, and 100 mg L^−1^ of Fe_3_O_4_).

The catalytic performance of the catalyst was tested under different experimental conditions. Table 1 summarizes the results of the 5-FU degradation, TOC conversion, and the corresponding concentration of iron species in the solution at the end of the experiments (60 min) in the solar photo-Fenton experiments for different pH values, catalyst loads, and H_2_O_2_ concentrations. Blank experiments at pH 3.0 were also performed in order to assess the influence of different factors on the degradation of 5-FU. In particular, Fe_3_O_4_/Solar (without H_2_O_2_ addition), H_2_O_2_ assisted photolysis (H_2_O_2_/Solar), homogeneous Fenton process (Fe_3_O_4_/H_2_O_2_), and homogeneous photo-Fenton process (FeSO_4_/H_2_O_2_/Solar) are presented in Table 2 and Figure 4 (Figure 4 also shows the heterogeneous photo-Fenton process, Fe_3_O_4_/H_2_O_2_/Solar for comparison purpose). The obtained results showed that: (i) 5-FU is stable in a water solution under solar radiation (photolysis); (ii) the amount of 5-FU degraded using Fe_3_O_4_ as a heterogeneous photocatalyst under solar radiation is also negligible (Fe_3_O_4_/solar); (iii) 5-FU is poorly removed by only H_2_O_2_ and solar radiation (H_2_O_2_/solar); and (iv) Fenton and photo-Fenton reactions are catalyzed by Fe_3_O_4_ and H_2_O_2_ (Fe_3_O_4_/H_2_O_2_), and in the presence of solar radiation (Fe_3_O_4_/H_2_O_2_/solar), respectively, with both 5-FU and TOC conversions being significantly increased (Figure 4). It is noteworthy that solar radiation not only improved the activity of the catalyst but also the selectivity of the process. Thus, although the heterogeneous Fenton-like process removed 80% of the initial 5-FU concentration, only 26% was mineralized according to TOC determinations (Table 1). However, in the case of the photo-Fenton process, 5-FU could be removed completely (100%), with a TOC removal of 76%. Nevertheless, the iron leaching under these conditions also increased from 0.80 to 1.97 mg L^−1^, which could compromise the viability of the processes.

There is a strong interest regarding the control of Fe_3_O_4_ nanoparticles, including band-gap features, in order to enhance the magnetite photoactivity. In this sense, plant-root extract, as both a precipitating and capping agent, is used to obtain spherical magnetite nanoclusters. The band-gap of materials is strongly dependent on the precipitation method, varying from 1.97 eV to 2.51 eV, when NaOH and NH_3_ · H_2_O are used as coprecipitating agents, respectively [34]. Magnetite nanoparticles with a BET surface of around 40 m^2^ g^−1^ were obtained with a very low band-gap of 1.2 eV, using the citrate sol-gel method. Nevertheless, this value can increase to around 3.0 eV, depending on the crystal size [32]. In this work, using a very simple coprecipitation method with NH_3_, we obtained a magnetite-based material, nanostructured as clusters of sphere-like nanoparticles, with a high mesoporosity and surface area, as well as a band-gap value of 1.57 eV and proved photocatalytic activity under solar radiation.

**Figure 5:**
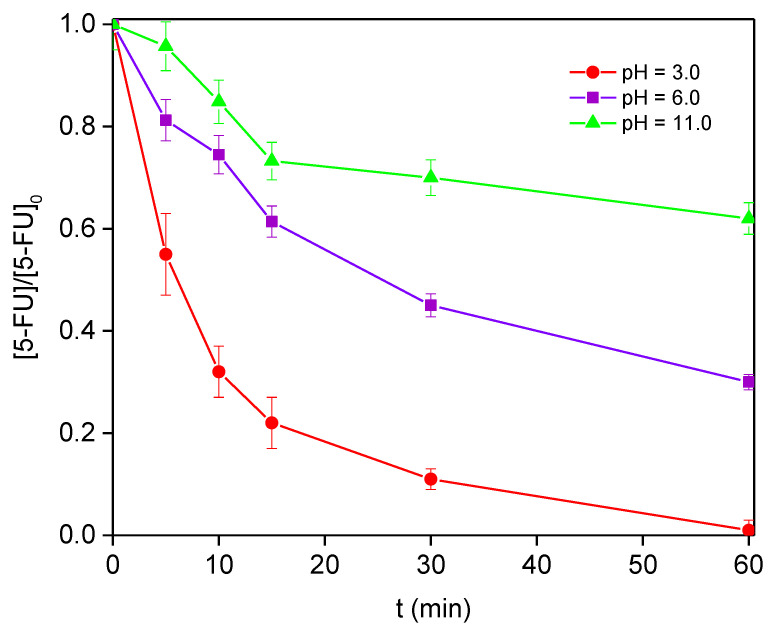
5-FU degradation at various pH values under simulated solar radiation (58 mM of H_2_O_2_ and 100 mg L^−1^ of Fe_3_O_4_).

Once the photoactivity of the synthesized nanoparticles in the Fenton reaction was proved, different experiments were carried out in order to assess the optimal operation conditions. The first parameter analyzed was the influence of the solution pH (Figure 5). As is typically observed for heterogeneous Fenton catalysts, the reaction rate decreased with increasing pH values [35]. At high values, the decomposition of hydrogen peroxide into water and oxygen occurs, leading to a reduction in the amount of oxidant available for the generation of the hydroxyl radicals.

Figure 5 shows the pH influence on the 5-FU conversion, the highest 5-FU conversion (100%) being achieved after 1 h of reaction under acidic conditions (pH = 3.0). When the solution pH was increased (up to 11.0), the 5-FU removal decreased to 70 and 38% for pH = 6.0 and 11.0, respectively. Similarly, the TOC removal decreased in the same trend, i.e., 76, 48, and 23% for pH = 3.0, 6.0, and 11.0, respectively. Thus, the ratio mineralization/total conversion is also favored with decreasing pH values and, thereby, it is expected that the reaction intermediates formed under basic conditions would be more recalcitrant. However, the acidity favors the dissolution of magnetite and, thus, the leaching detected was 1.97 mg L^−1^ at pH = 3.0 vs. 1.00 mg L^−1^ at pH = 11.0, influencing the catalyst stability. Moreover, the leaching of metal ions into the solution has to be avoided to prevent additional contamination and to respect environmental regulations (maximum of 2 mg L^−1^ in EU directives (EEC List of Council Directives 76/4647. European Economic Community; Brussels, 1982)). Under our experimental and acidic conditions, the leaching degree was close to the established limit. That concentration of iron in the solution could present a significant contribution of the Fenton reaction in the homogeneous phase. In order to determine the influence on the results shown in Figure 4, additional experiments with an Fe^2+^ concentration of around 2 mg L^−1^ (equivalent to the amount leached) were performed, using FeSO_4_ solutions at pH = 3.0 (FeSO_4_/H_2_O_2_/Solar, Table 2 and Figure S1, Supplementary Information). The 5-FU conversion after 1 h of reaction reached 42%, although the catalyst seemed to be deactivated and/or the reaction intermediates were more recalcitrant, because the 5-FU conversion was maintained after 30 min of reaction. In addition, iron species in the solution could interact with reactants or intermediates.

**Figure 6:**
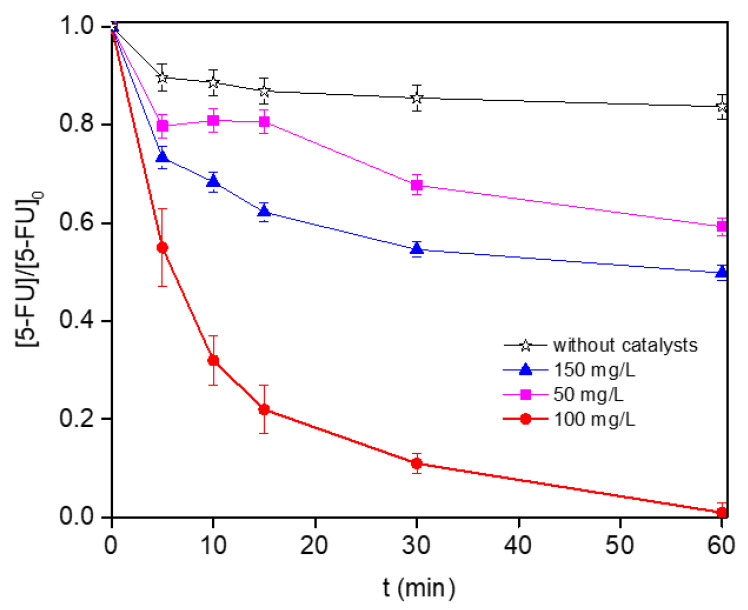
Influence of the catalyst loading on the 5-FU degradation under simulated solar radiation.

The catalyst loading is another important parameter, determining not only the process cost, but rather the environmental impact in terms of metal leaching because both parameters increased simultaneously. The effects of catalyst load on the 5-FU conversion, at a fixed H_2_O_2_ dosage (58 mM) and pH = 3.0, are shown in Figure 6. The pollutant conversion and mineralization removal strongly increased with an increase in the catalyst loading from 50 to 100 mg L^−1^, but both of them decayed using 150 mg L^−1^ (Table 1). The overloading effect can be associated with the formation of agglomerates in suspension, limiting the accessibility/diffusion of reactants/products with the formation of dark zones inaccessible to radiation, which would make the photoactivation of the active sites difficult. It is noteworthy that the presence of the catalyst plays a significant role in the process efficiency, since the TOC removal was around 6% for the experiment carried out in the absence of catalyst.

**Figure 7:**
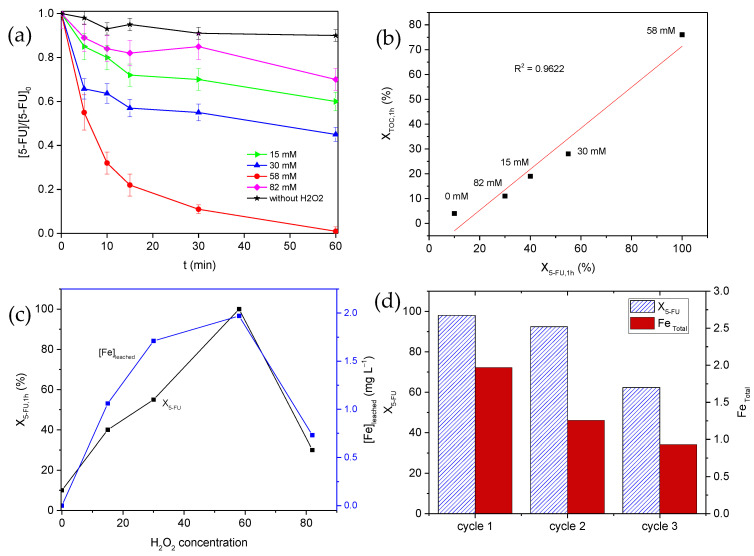
(**a**) Effects of the H_2_O_2_ concentration on the 5-FU degradation under simulated solar radiation; (**b**) correlation between the 5-FU conversion (X_5-FU_) and the mineralization degree achieved in 60 min; (**c**) relationship between X_5-FU_ and Fe-leaching with the H_2_O_2_ concentration (pH = 3.0 and 100 mg L^−1^ Fe_3_O_4_); and (**d**) X_5-FU_ and iron leached with the catalyst reusability during three consecutive cycles.

The influence of the H_2_O_2_ concentration on the development reaction is shown in Figure 7a. The experiments were performed at different H_2_O_2_ dosages i.e., 15, 30, 58, and 82 mM. An increase in the hydrogen peroxide concentration (up to 58 mM) triggers a higher 5-FU degradation due to more HO^•^ radical generation. These radicals could attack the contaminant molecules, reaching degradations of 31%, 52%, and 100% for 15, 30, and 58 mM, respectively. On the contrary, at the highest H_2_O_2_ concentration (i.e., 82 mM), a decrease in 5-FU and TOC conversion, reaching 30 and 11%, respectively, was observed. This fact is explained by the possible inhibiting effect of the high hydrogen peroxide concentration because it acts as a HO^•^ radical scavenger, reducing their presence and, consequently, the attack on the contaminant molecules (Equation (4)). In this way, hydroperoxyl radicals with lower oxidative potential are generated. In addition, these radicals are also able to react with hydroxyl radicals to form oxygen and water, diminishing their amount and the 5-FU degradation (Equation (5)) [36].

HO^•^ + H_2_O_2_ → HO_2_^•^ + H_2_O (4)

HO_2_^•^ + HO^•^ → H_2_O + O_2_ (5)

In spite of these adverse reactions, the relationship of the total 5-FU removal regarding the mineralization degree was analyzed (Figure 7b). The results show a linear correlation, which confirms that the reaction progress follows a similar mechanism and that the proportion of products is similar, independent of the H_2_O_2_ concentration. Although, evidently, scavenging reactions lead to H_2_O_2_ consumption without any advance and, consequently, to a higher process cost.

Another relevant aspect to be considered is the stability of the catalyst. Figure 7c shows that the 5-FU conversion (*X*_5-FU_) follows the same trend as the iron leached (Fe-_leached_) with the H_2_O_2_ dosage. This behavior seems to be related to the formation of iron complexes on the catalyst surface. These complexes have a photolabile nature, so when they are irradiated, they can be dissociated into their ions (Equation 6), which also enhances the homogeneous component of the process and does not occur in the Fenton process, where they are stable in the aqueous medium [37,38]. In this photodegradation, a superior number of hydroxyl radicals should be generated, which is another reason for the enhanced 5-FU degradation efficiency during the solar photo-Fenton process, compared to the conventional Fenton process without radiation (Figure 5). Among the iron species, the most active one is [Fe(OH)]^2+^, which mostly presents at acidic pHs (Equation (6)) [39,40].

[Fe(OH)]^2+^ + hν → Fe^2+^ + HO^•^ (6)

Once the different operational parameters were studied, the optimal conditions were established as acidic pH = 3.0, 58 mM of H_2_O_2_, 100 mg L^−1^ of catalyst load, and using simulated solar radiation (solar photo-Fenton process). The reusability of the catalyst during consecutive degradation cycles was then analyzed. For this purpose, three consecutive reaction runs were carried out with the same methodology already described. The results obtained are summarized in Figure 7d. As commented along the manuscript, the main cause of the catalyst deactivation can be associated to the leaching degree. This parameter was significantly high during the first cycle, favoring the homogenous component of the reaction (Figure S1, Supplementary Information); however, the activity of the catalyst surface remained practically unchanged. On the contrary, the lixiviation was moderate during the second cycle, but deactivation was stronger after this second run. Therefore, it is reasonable to conclude that the catalyst can be used with efficiency at least twice, still maintaining most of its characteristics, while its use during another consecutive cycle leads to an important reduction in the degradation.

## 4. Conclusions

Spherical nanoparticles of mainly the Fe_3_O_4_ magnetite phase were obtained through a simple coprecipitation method by adjusting the ratio of Fe^2+^/Fe^3+^ and a controlled basification with NH_3_. The clusters of formed nanoparticles provided a low BET surface area due to the absence of micropores but a high mesoporosity, as a consequence of the interstitial voids of nanoparticles. The degradation of the 5-FU antitumoral drug by using H_2_O_2_ as an oxidant agent was studied, and the heterogeneous Fenton reaction was compared with the photo-Fenton process. Furthermore, the effects of pH, catalyst loading, and H_2_O_2_ concentration on the total 5-FU removal and the degree of mineralization were analyzed. The optimal conditions were established, avoiding the excess of catalyst and H_2_O_2_, which had a detrimental effect on the overall catalyst efficiency due to a scavenging effect. The optimal conditions were an acidic pH of 3.0, 58 mM of H_2_O_2_, and 100 mg L^−1^ of catalyst load under simulated solar radiation (solar photo-Fenton process), with a total 5-FU removal and high mineralization degree (76%) being achieved. The contribution of iron leaching to the catalyst deactivation, the role of the dissolved iron ions on homogenous reactions, and the stability of the catalyst were pointed out during consecutive reaction cycles. A reasonable performance was obtained at least twice but with a leached iron that was always lower than that recommended by the environmental regulations.

## Figures and Tables

**Table 1:** Different experimental conditions used in the solar photo-Fenton experiments for the 5-FU degradation and respective Fe-leached and TOC conversion after 1 h of reaction.

Parameter	pH	Catalyst Load (mg L^−1^)	H_2_O_2_ (mM)	X_5-FU,1h_ (%)	X_TOC,1h_ (%)	Fe-_leached_ (mg L^−1^)
**pH value**	**3.0**	100	58	100	76	1.97
	**6.0**	100	58	70	48	0.40
	**11.0**	100	58	38	23	1.00
**Catalyst load**	3.0	**0**	58	16	6	-
	3.0	**50**	58	41	28	0.27
	3.0	**100**	58	100	76	1.97
	3.0	**150**	58	50.2	30	2.10
**H_2_O_2_ concentration**	3.0	100	**0**	10	4	-
	3.0	100	**15**	40	19	1.06
	3.0	100	**30**	55	28	1.71
	3.0	100	**58**	100	76	1.97
	3.0	100	**82**	30	11	0.73

**Table 2:** 5-FU conversion (X_5-FU,1h_), total organic carbon conversion (X_TOC,1h_), and iron-leached (Fe-_leached_) for the blank experiments.

Blank Experiments	pH	Catalyst Load (mg L^−1^)	H_2_O_2_ (mM)	X_5-FU,1h_ (%)	X_TOC,1h_ (%)	Fe-_leached_ (mg L^−1^)
**Fe_3_O_4_/Solar**	3.0	100	0	10	4	0.5
**H_2_O_2_/Solar**	3.0	0	58	16	6	-
**Fe_3_O_4_/H_2_O_2_**	3.0	100	58	80	26	0.8
**FeSO_4_/H_2_O_2_/Solar**	3.0	100	58	42	30	-

## Supplementary Materials

The following are available online at https://www.mdpi.com/article/10.3390/nano12244438/s1, Figure S1: Influence of the homogeneous process on the 5-FU degradation under simulated solar radiation (58 mM H_2_O_2_ pH = 3.0 and 2 mg L^−1^ FeSO_4_) [17,41,42,43,44,45,46,47].
