# Supplementary material for: Magnetite Nanoparticles as Solar Photo-Fenton Catalysts for the Degradation of the 5-Fluorouracil Cytostatic Drug
Source: Nanomaterials (Basel). 2022 Dec 13;12(24):4438. doi: 10.3390/nano12244438 (PMC9782202; doi:10.3390/nano12244438)
Supplement: Supplementary file 1 [file nanomaterials-12-04438-s001.zip › nanomaterials-2079622-supplementary.pdf]

Supplementary Information

Magnetite Nanoparticles as Solar Photo-Fenton Catalysts for the Degradation of the 5-Fluorouracil Cytostatic Drug

Lorena T. Pérez-Poyatos, Sergio Morales-Torres, Francisco J. Maldonado-Hódar and Luisa M. Pastrana-Martínez *

NanoTech—Nanomaterials and Sustainable Chemical Technologies, Department of Inorganic Chemistry, Faculty of Sciences, University of Granada, ES18071 Granada, Spain

* Correspondence: lpastrana@ugr.es; Tel.: +34-958248489

2.1 Chemicals

Iron(III) chloride hexahydrate ($FeCl_3{\cdot}6H_2O$, > 99%), iron(II) chloride tetrahydrate, ($FeCl_2{\cdot}4H_2O$, 98%), sodium hydroxide (NaOH, > 99%), potassium hydroxide (KOH, > 90%), sulfuric acid (H_2SO_4, ≥ 99.7%), H_2O_2 (30% w/w) and 1,10-phenanthroline ($C_{12}H_8N_2$, > 99%), were obtained from Sigma-Aldrich. Iron(II) sulfate heptahydrate ($FeSO_4{\cdot}7H_2O$, > 99.5%) was supplied by Merck, while 5-FU ($C_4H_3FN_2O_2$, >99%) was purchased from Tokyo Chemical Industry.

2.2 Synthesis of Fe_3O_4 nanoparticles

During the synthesis of the iron (II, III) oxide (Fe_3O_4) magnetic nanoparticles, 12 g of $FeCl_3{\cdot}6H_2O$ was dissolved in 50 mL of distilled water. Then, 6 g of $FeCl_2{\cdot}4H_2O$ was also dissolved in 50 mL of distilled water with 0.2% of HCl, to avoid the precipitation of Fe^{3+} hydroxides and the possible oxidation of Fe^{2+}. These quantities represent a $[Fe^{3+}]/[Fe^{2+}]$ ratio of 1.5. Once all the iron salts were dissolved, both solutions were mixed and the resultant solution was vigorous stirring during 10 min. Thereafter, 25 mL of NH_4OH at 25% was added slowly to the mixture obtained and stirred for 10 min at room temperature, resulting in a black precipitate. The synthesized solid was recovered magnetically, washed with abundant distilled water and dried in an oven at 120 °C for 12 h. The final material was grinded, obtaining a 70% of yield. The sample was labelled as Fe_3O_4.

2.3 Characterization techniques

The synthesized catalyst was characterized by N_2 adsorption-desorption isotherms at – 196 °C using a Quadrasorb SI equipment from Quantachrome. Brunauer-Emmett-Teller (BET) equation was applied to calculate the apparent surface area (S_{BET}) [1,2], while the pore size distribution (PSD) was obtained by Barret-Joyner-Hallender (BJH) method [3]. The total pore volume of the samples (V_T) was calculated as the volume of N_2 adsorbed at $P/P_0 = 0.95$. The morphology of the prepared materials was studied by scanning electron microscopy (SEM) using a LEO (Carl Zeiss) GEMINI-1430VP microscope. Transmission electron microscopy (HRTEM) images were taken using a FEI Titan G2 60–300 microscope with a high brightness electron gun (X-FEG) operated at 300 kV and equipped with a Cs image corrector (CEOS). The X-ray diffraction (XRD) patterns were recorded using a Philips PW 1710 diffractometer (Bruker, Rivas-Vacia, Madrid, Spain), using the CuKα radiation and a nickel filter that removes the κβ radiation. The average crystal size (d_{Fe3O4}) of the materials was calculated by applying the Scherrer equation [4]. The point of zero charge pH (pH_{PZC}) was determined by a pH drift method [5,6]. The optical properties of catalysts were analyzed by UV-Vis spectrophotometer CARY 5E from VARIAN equipped with a diffuse reflectance accessory (DRA). The band gap of the samples was determined from the corresponding Tauc's plots using Kubelka-Munk units $(K{\cdot}M{\cdot}E)^{1/2}$ as a function of energy (eV).

2.4 Degradation study by solar-photoFenton process

In a typical run, a catalyst load of 100 mg L^{-1} was added to 60 mL of the 5-FU solution in a glass cylindrical reactor and magnetically stirred in darkness for an hour to reach the adsorption-desorption equilibrium. Once the equilibrium was achieved, the Solarbox was turned-on and H_2O_2 simultaneously added (time zero for reaction). The experiments were done maintaining a constant air flow through the solution, under continuous stirring and room temperature (25 °C). Samples were withdrawn at regular time intervals and filtered with a syringe filter made of polyethersulfone (PES) with a 0.45 μm pore size, to remove the solid catalyst.

The 5-FU concentration was measured by using an Ultra High Performance Liquid Chromatography (UHPLC), with a Shimadzu Corporation apparatus (Nexera model, Tokyo, Japan) equipped with a Shimpack GISS-HP C18, 3 μm column (100 x 3.0 mm I.D.) and a Diode Array Detector. The injection volume was 20 μL and the mobile phase consisted of a mixture of water and methanol (97:3 v/v) at isocratic mode with a flow rate of 0.2 mL min^{-1}. Concentrations of Fe^{2+} and Fe^{3+} were measured at the end of selected experiments using a spectrophotometric method using 1,10-phenanthroline as reagent and measuring the absorbance at 510 nm [7,8]. H_2O_2 concentration was determined by adding 1 mL of a 0.5 M H_2SO_4 solution and 0.1 mL of $TiO(SO_4)$ (15 wt.% in diluted H_2SO_4) to 1 mL of the liquid sample, and measuring the respective absorbance at 405 nm in a Shimadzu UV-2600 spectrophotometer. Total organic carbon (TOC) of initial and final samples was determined for selected samples at the end of the experiments using a Shimadzu TOC-5000A analyzer.

The synthesized catalyst was reused in various consecutive cycles with the optimized conditions previously established. For this purpose, once the reaction was finished, the catalyst was removed with the help of a magnet, washed with DI water and dried in an oven at 100 °C and reused in consecutive cycles maintaining the catalyst loading (100 mg L^{-1}) and the rest of operational conditions.

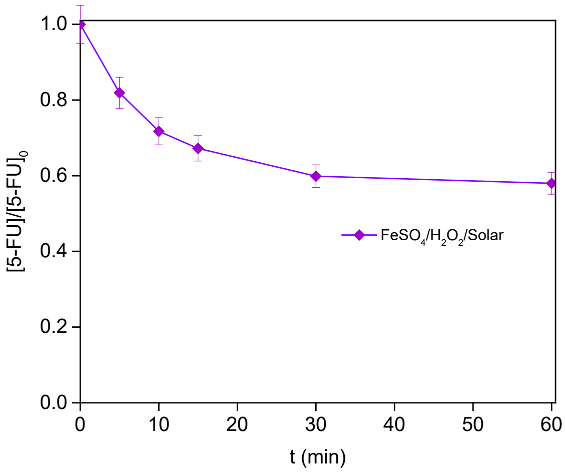

Figure S1. Influence of the homogeneous process on the 5-FU degradation under simulated solar radiation (58 mM H_2O_2 pH= 3.0 and 2 mg L^{-1} $FeSO_4$).

References

1. Brunauer, S.; Emmett, P.H.; Teller, E. Adsorption of gases in multimolecular layers. *Journal of the American Chemical Society* **1938**, *60*, 309-319,
2. K.S.W. Sing, D.H.E., R.A.W. Haul, L. Moscou, R.A. Pierotti, J. Rouquérol, T. Siemieniewska. Reporting physisorption data for gas/solid systems with special reference to the determination of surface area and porosity. *International Union of Pure and Applied Chemistry* **1985**, *57*, 603 - 619,
3. Barrett, E.P.; Joyner, L.G.; Halenda, P.P. The determination of pore volume and area distributions in porous substances. I. Computations from nitrogen isotherms. *Journal of the American Chemical Society* **1951**, *73*, 373-380,
4. Cullity, B.D.; Stock, S.R. *Elements of X-ray Diffraction, Third Edition*. Prentice-Hall: New York, 2001.
5. Newcombe, G.; Hayes, R.; Drikas, M. Granular activated carbon: Importance of surface properties in the adsorption of naturally occurring organics. *Colloids and Surfaces A: Physicochemical and Engineering Aspects* **1993**, *78*, 65-71, doi: 10.1016/0927-7757(93)80311-2.

6. Pastrana-Martínez, L.M.; Morales-Torres, S.; Carabineiro, S.A.C.; Buijnsters, J.G.; Figueiredo, J.L.; Silva, A.M.T.; Faria, J.L. Photocatalytic activity of functionalized nanodiamond-TiO_2 composites towards water pollutants degradation under UV/Vis irradiation. *Applied Surface Science* **2018**, *458*, 839-848, doi: 10.1016/j.apsusc.2018.07.102.
7. ISO 6332:1988. Water Quality: Determination of iron. Spectrometric method using 1,10-phenanthroline.
8. Pastrana-Martínez, L.M.; Pereira, N.; Lima, R.; Faria, J.L.; Gomes, H.T.; Silva, A.M.T. Degradation of diphenhydramine by photo-Fenton using magnetically recoverable iron oxide nanoparticles as catalyst. *Chemical Engineering Journal* **2015**, *261*, 45-52, doi: 10.1016/j.cej.2014.04.117.
